# AGE-RELATED DEFICITS IN MOBILITY RESILIENCE WITH CLIMATE CHANGE: A CASE STUDY OF HURRICANE IAN

**DOI:** 10.1093/geroni/igae098.0436

**Published:** 2024-12-31

**Authors:** Todd Manini, Shangde gao, Yan Wang

**Affiliations:** University of Florida College of Medicine, Gainesville, Florida, United States; University of Florida, Gainesville, Florida, United States; University of Florida, Gainesville, Florida, United States

## Abstract

Resilience is the capacity of a system to respond and recover from extreme events. We aimed to test the feasibility of a new metric of mobility resilience in the context of an extreme hurricane event. We used the concept of the resilience triangle — an engineering framework to conceptualize the resilience of physical infrastructure systems in the public domain. Mobility measures originate from point of interest (POI) visit data using geolocations of 47 million smartphone devices across the U.S.— about 10% of the population in the nation. These data were linked to 4,749 exposed neighborhoods (i.e. census tracts) within Florida who were impacted by Hurricane Ian in 2022. POI data were tracked 1 month prior, and 12 months following hurricane Ian to quantify the impact (ratio of pre-event mobility to maximal loss of mobility), recovery (ratio of post-event to pre-event mobility equilibrium), and duration of recovery (length of time from nadir of mobility lost to a new mobility equilibrium). In multiple regression models, older neighborhood median age was associated with worse recovery and longer duration of recovery. Additionally, neighborhoods with a higher percentage of adults 65+ years had a higher impact, worse recovery, and longer duration of recovery. Adjustments for wind speed, socioeconomic status (poverty, group housing, house cost/structure, uninsured rates), physical/mental limitations, mobile home dwellings, and racial/ethnic minorities had little effect on associations. This study underscores the potential of resilience triangle framework for measuring mobility resilience, although more research across diverse climate events and locations is necessary.

